# Supplementary material for: Modeling confinement and reversibility of threshold-dependent gene drive systems in spatially-explicit Aedes aegypti populations
Source: BMC Biol. 2020 May 12;18:50. doi: 10.1186/s12915-020-0759-9 (PMC7218562; doi:10.1186/s12915-020-0759-9)
Supplement: Supplementary file 1 — Additional file 1: Table S1. Life history, population size and movement parameters for Aedes aegypti in Cairns, Australia. [file 12915_2020_759_MOESM1_ESM.docx]

**Table S1. Life history, population size and movement parameters for *Aedes aegypti* in Cairns, Australia.**

| **Parameter** | **Symbol** | **Value** | **Reference** |
| --- | --- | --- | --- |
| Egg production per female (day^-1^) | *β* | 20 | [(30)](https://paperpile.com/c/5q4L6l/p1ie) |
| Duration of egg stage (days) | *T_E_* | 2 | [(31)](https://paperpile.com/c/5q4L6l/K73p) |
| Duration of larval stage (days) | *T_L_* | 5 | [(31)](https://paperpile.com/c/5q4L6l/K73p) |
| Duration of pupa stage (days) | *T_P_* | 1 | [(31)](https://paperpile.com/c/5q4L6l/K73p) |
| Daily population growth rate (day^-1^) | *r* | 1.175 | [(32)](https://paperpile.com/c/5q4L6l/eZo5) |
| Daily mortality rate of adult stage (day^-1^) | *μ_M_* | 0.090 | [(33–35)](https://paperpile.com/c/5q4L6l/G9XZ+4mBQ+0wsR) |
| Adult population size per household | *N_H_* | 15 | [(22)](https://paperpile.com/c/5q4L6l/OWtQ) |
| Daily probability adult leaves household | *p* | 0.28 | [(36, 37)](https://paperpile.com/c/5q4L6l/stT1+JqA5) |
| Mean dispersal distance of adult conditional upon movement (meters) | *d* | 54.1 | [(38)](https://paperpile.com/c/5q4L6l/kiUW) |
