# Supplementary figures and images for: Modeling confinement and reversibility of threshold-dependent gene drive systems in spatially-explicit Aedes aegypti populations
Source: BMC Biol. 2020 May 12;18:50. doi: 10.1186/s12915-020-0759-9 (PMC7218562; doi:10.1186/s12915-020-0759-9)

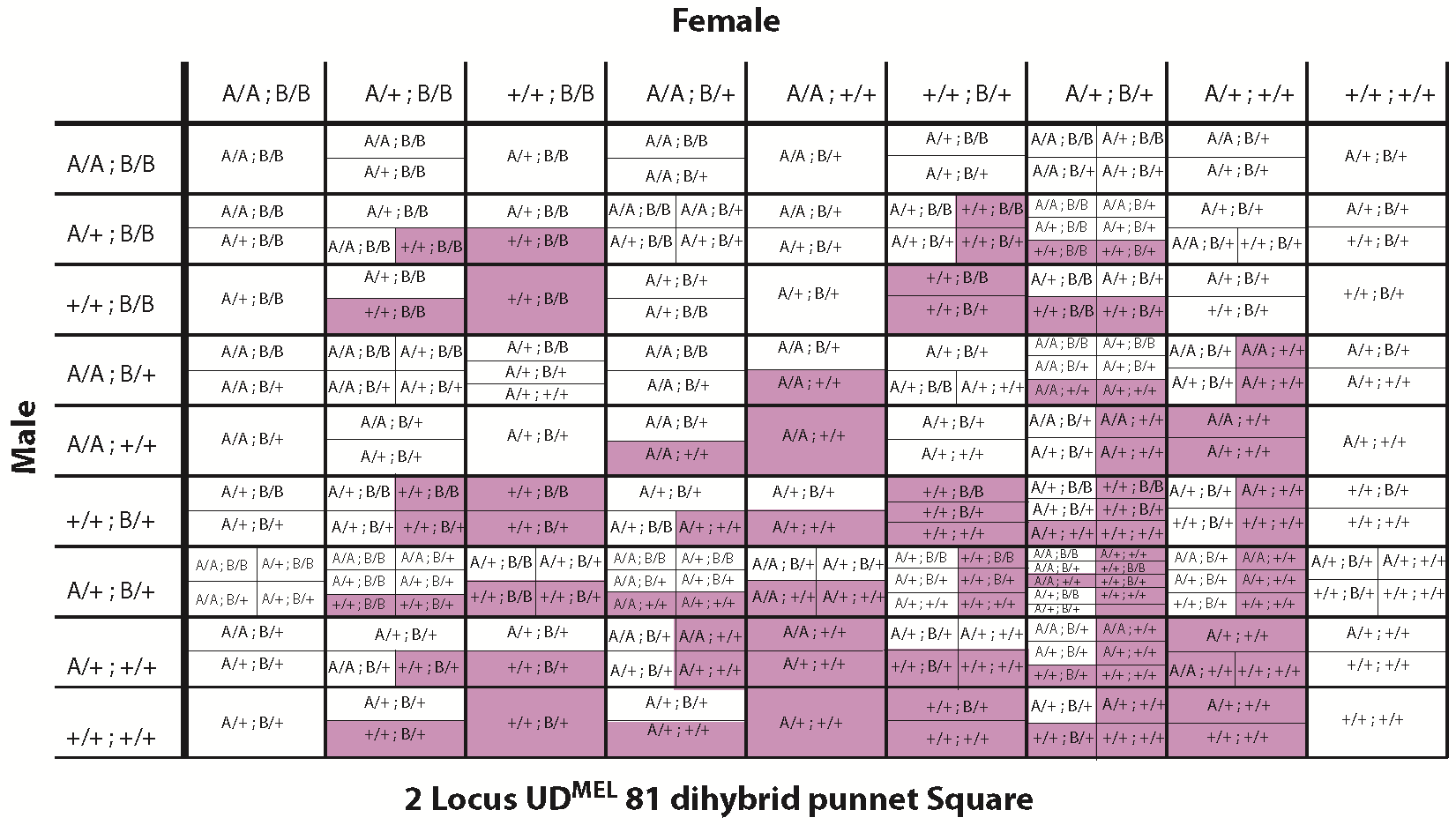

Supplement: Supplementary file 2 — Additional file 2: Figure S1. Complete inheritance pattern of UDMEL. UDMEL is composed of two unlinked constructs (here referred to as A and B), each consisting of a maternally-expressed toxin and a zygotically-expressed antidote for the toxin on the opposite construct (see Fig. 1b). The cross here represents matings between all nine possible parental genotypes (“+” represents the wild-type allele, and “A” and “B” represent alleles corresponding to the two UDMEL constructs). Offspring lacking the antidotes to the maternal toxins produced by their mother are unviable (shaded). At high population frequencies, the selective advantage on the constructs, by virtue of the antidotes, outweighs the fitness load due to the toxins, and hence results in frequency-dependent spread. [file 12915_2020_759_MOESM2_ESM.tiff]

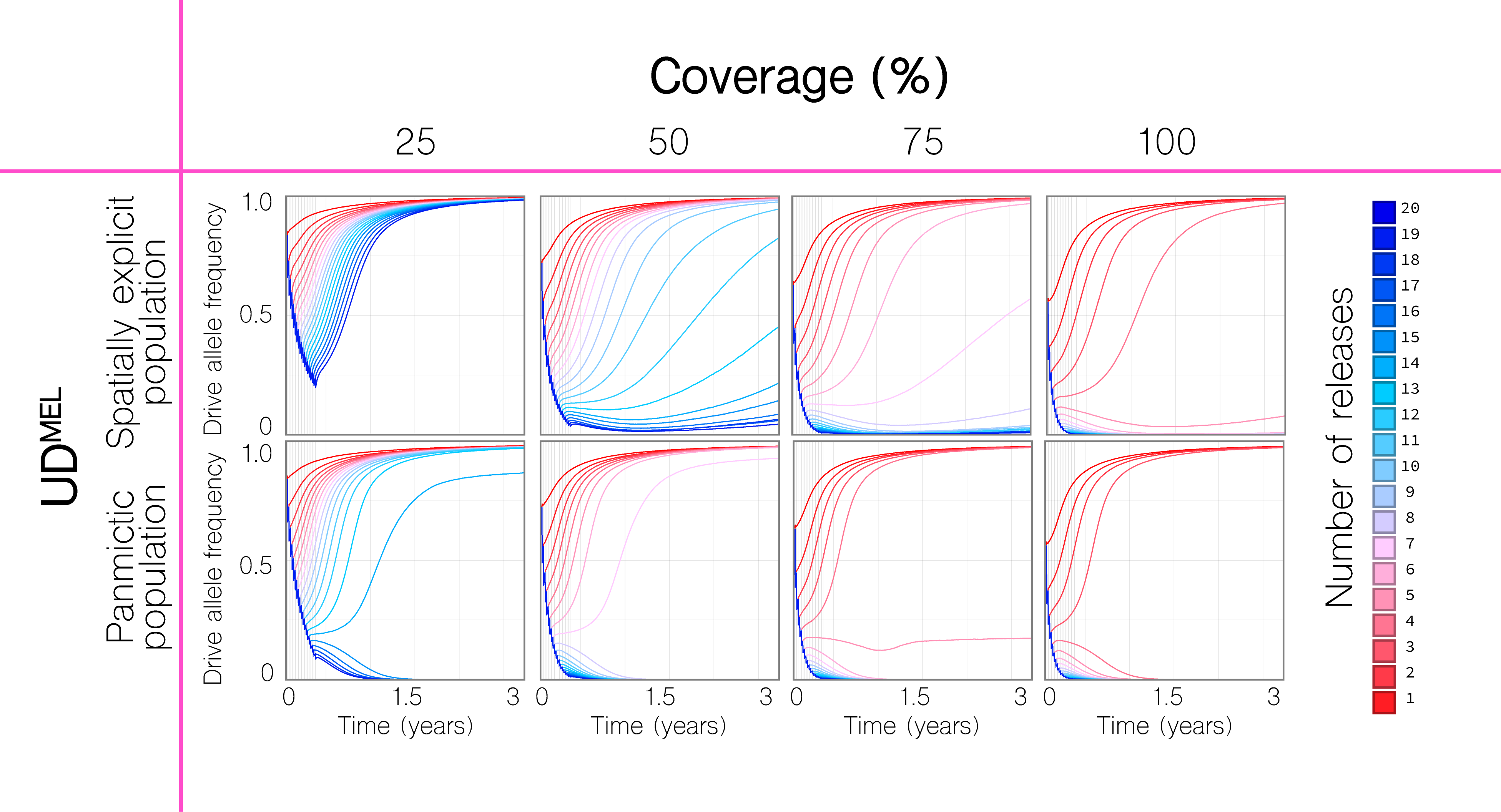

Supplement: Supplementary file 4 — Additional file 4: Figure S3. Remediation results for UDMEL in spatially-explicit and panmictic populations. Time-series results are shown for a given number of weekly releases of 20 adult wild-type Ae. aegypti per household (10 female and 10 male) with the intent of remediation in the community of Yorkeys Knob (Fig. 1c), and at given coverage levels, where coverage is the proportion of households that receive the releases. (Top) Remediation in the spatially-explicit population is extremely difficult. At a coverage level of 75%, nine weekly releases are required for a reduction in UDMEL allele frequency over the first year; however, complete remediation is not possible even with 20 releases. Complete remediation is possible at a coverage level of 100% for 15 or more weekly releases. (Bottom) Remediation in the panmictic population is much less demanding. At a coverage level of 25%, it can be achieved with 16 or more releases, and at a coverage level of 50%, it can be achieved with nine or more releases. [file 12915_2020_759_MOESM4_ESM.tif]

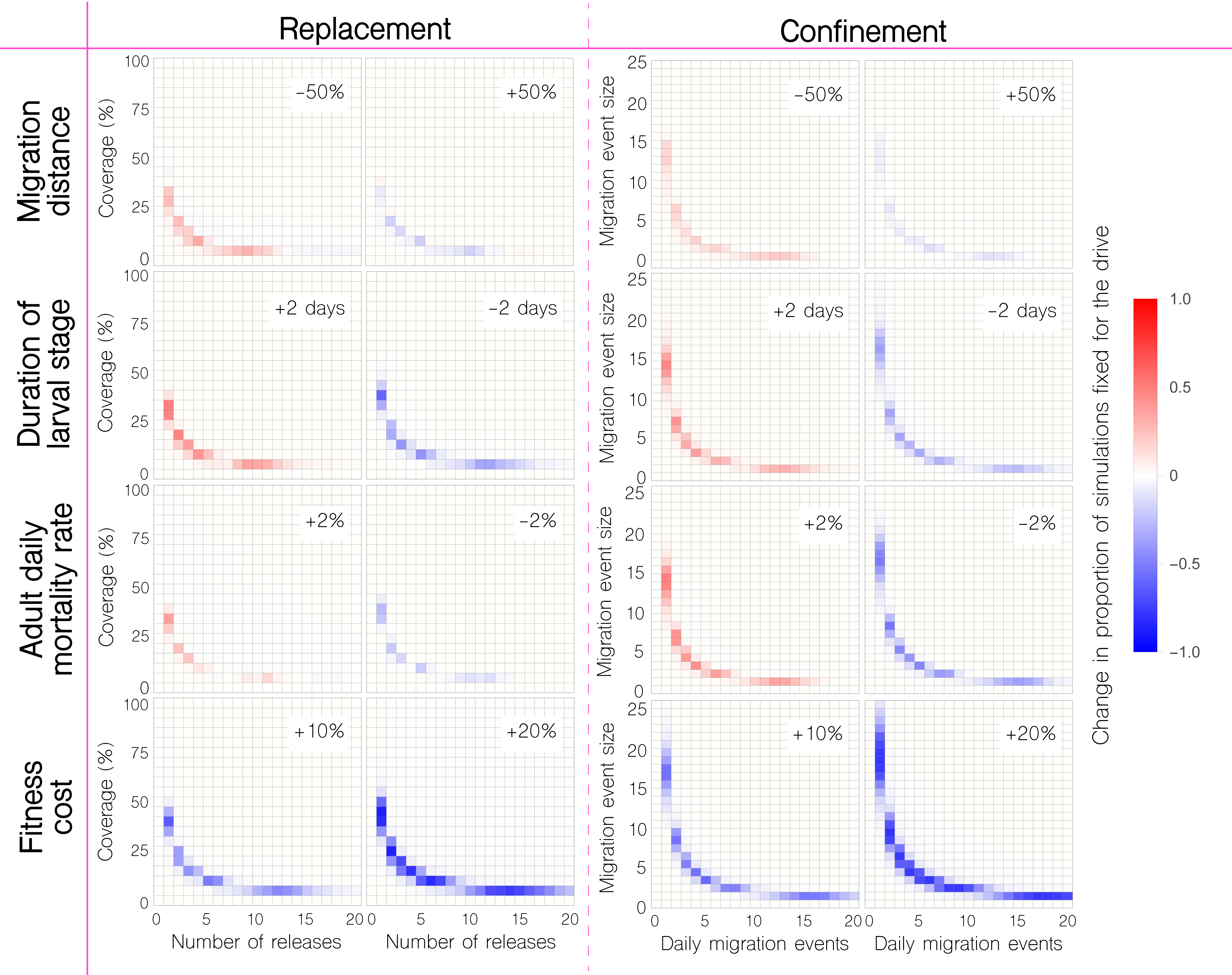

Supplement: Supplementary file 5 — Additional file 5: Figure S4. Sensitivity of model outcomes for replacement and confinement of UDMEL. Changes are depicted in the proportion of 50 stochastic simulations that result in fixation for replacement and confinement of UDMEL. Proportions are compared to those in the second row of Fig. 4 as we vary: i) the mean dispersal distance of adult mosquitoes (+/- 50%), ii) the duration of the larval life stage (+/- 2 days), iii) the baseline adult mortality rate (+/- 2%), and iv) the fitness cost associated with being homozygous for the translocation (+10% or +20%). UDMEL displays similar parameter sensitivities regarding fixation and batch migration outcomes as for translocations (Fig. 5), with the exception that these outcomes are less sensitive to fitness costs, likely due to the fact that fitness is accommodated through a reduction in female fecundity rather than an increase in adult mortality. [file 12915_2020_759_MOESM5_ESM.tif]

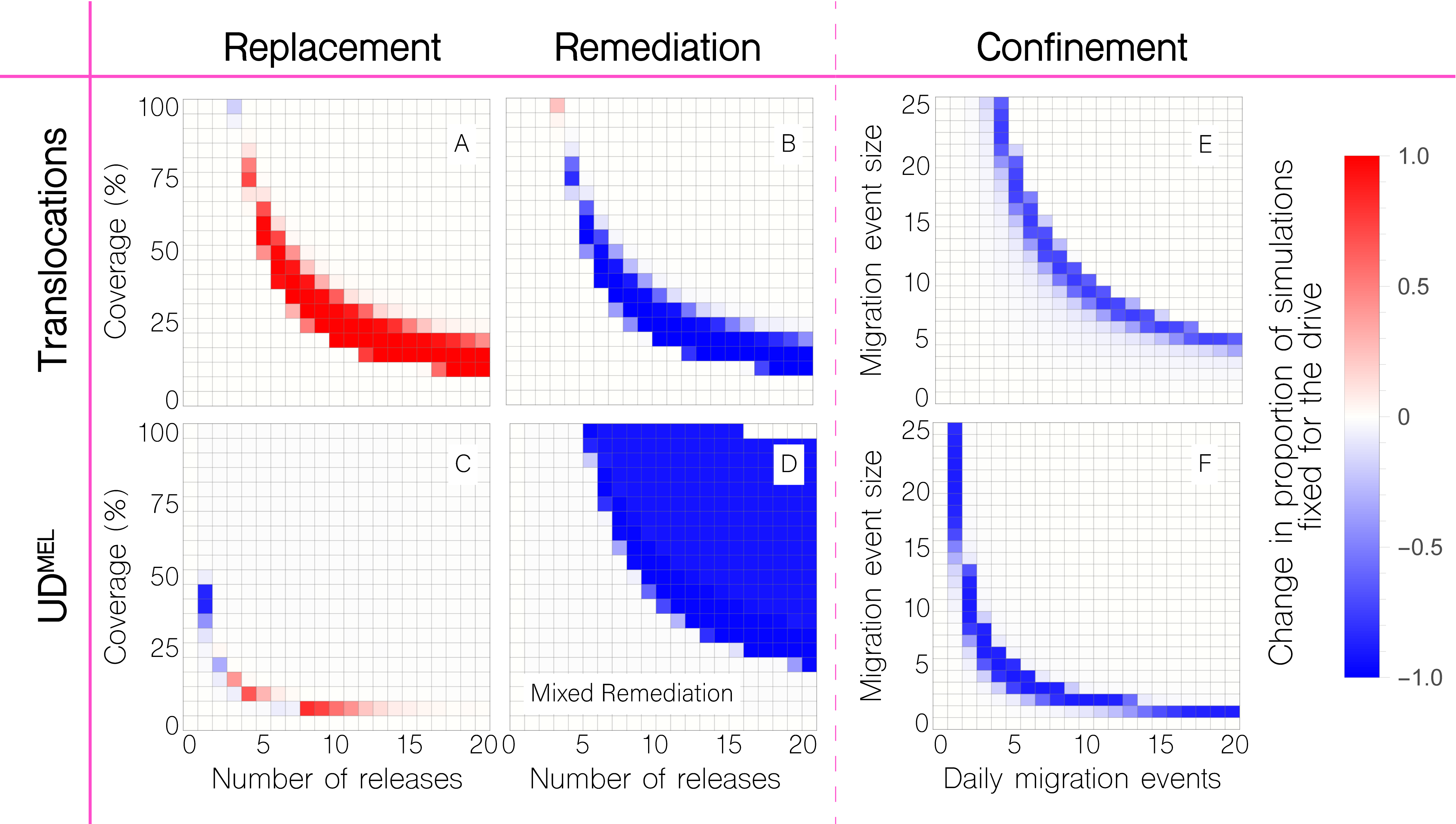

Supplement: Supplementary file 6 — Additional file 6: Figure S5. Sensitivity of model outcomes for translocations and UDMEL comparing spatially-explicit and panmictic populations. Changes are depicted in the proportion of 50 stochastic simulations that result in fixation for replacement, remediation and confinement of translocations and UDMEL. Proportions are compared to those in Fig. 4 as we simulate a model where Yorkeys Knob and Trinity Park are panmictic populations of equivalent size to their spatially-explicit versions. Introducing population structure greatly increases the release requirements to remediate UDMEL (as seen in Additional file 4: Fig. S3), and substantially increases the release requirements for replacement or remediation of translocations. Invasion of a neighboring population, on the other hand, requires moderately fewer daily migration events in structured populations for both translocation and UDMEL. [file 12915_2020_759_MOESM6_ESM.tif]

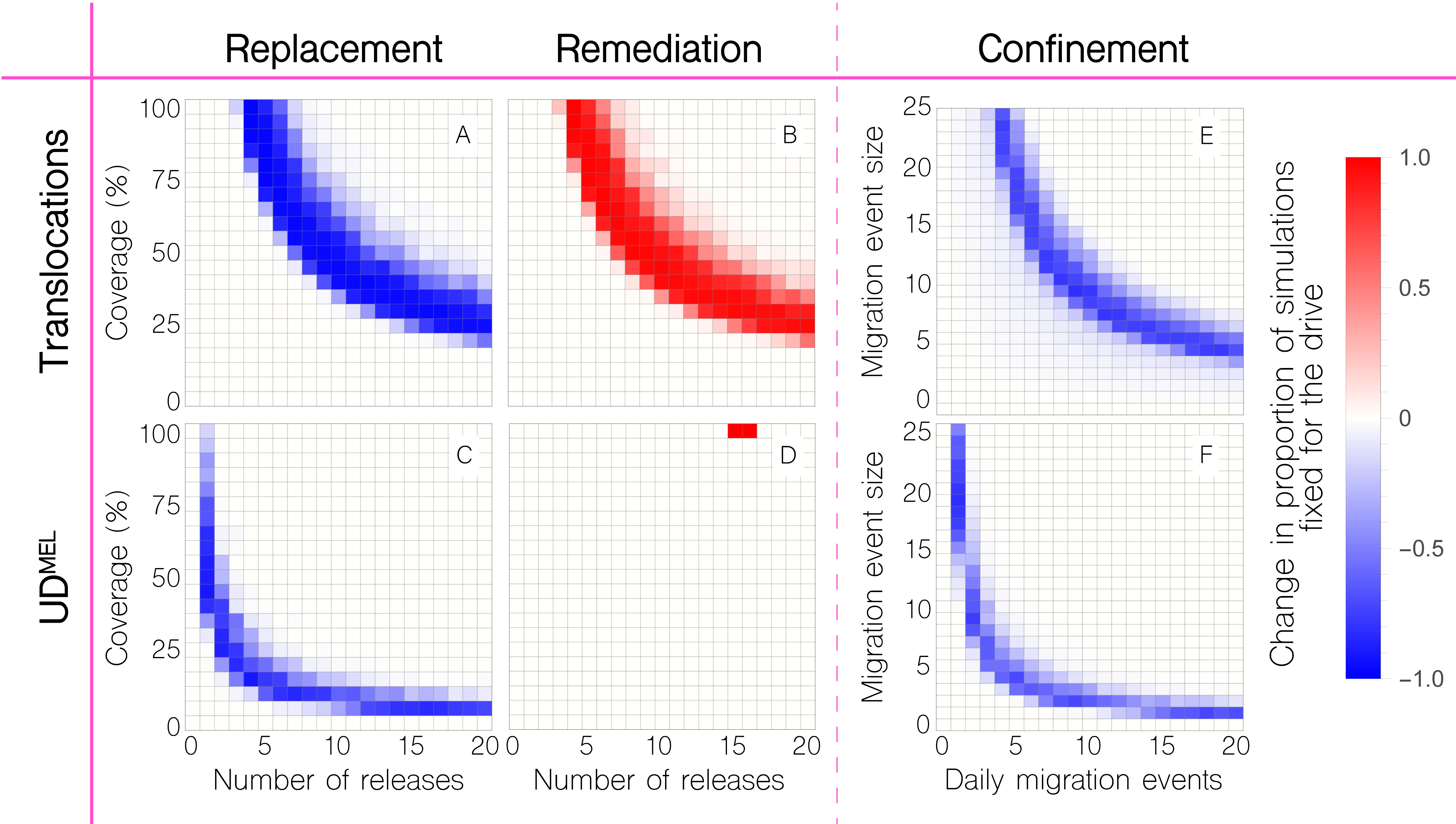

Supplement: Supplementary file 7 — Additional file 7: Figure S6. Sensitivity of model outcomes for translocations and UDMEL comparing spatially-explicit populations with and without heterogeneity in household mosquito population size. Changes are depicted in the proportion of 50 stochastic simulations that result in fixation for replacement, remediation and confinement of translocations and UDMEL. Proportions are compared to those in Fig. 4 as we simulate a model where household mosquito population size is distributed according to a zero-inflated, truncated exponential distribution with a mean of 15 adults, 55% of households having no mosquitoes, and none having more than 45 adults. Introducing household population size heterogeneity substantially increases release requirements for replacement and remediation with translocations, and marginally increases release requirements for replacement with UDMEL. Fortunately, population size heterogeneity makes confinement moderately more promising for both systems. [file 12915_2020_759_MOESM7_ESM.tif]
